# Optimizing access to fruits and vegetables in rural communities: A decision-making model for the placement of produce markets

**DOI:** 10.1371/journal.pone.0331545

**Published:** 2025-09-26

**Authors:** Eduardo Pérez, Cassandra M. Johnson, Yan Li, José A. Pagán

**Affiliations:** 1 Ingram School of Engineering, Texas State University, San Marcos, Texas, United States of America; 2 School of Family and Consumer Sciences, Texas State University, San Marcos, Texas, United States of America; 3 School of Public Health, Shanghai Jiao Tong University School of Medicine, Shanghai, China; 4 Department of Population Health Science and Policy, Icahn School of Medicine at Mount Sinai, New York, New York, United States of America; 5 Department of Public Health Policy and Management, School of Global Public Health, New York University, New York, United States of America; University of Georgia, UNITED STATES OF AMERICA

## Abstract

Many rural communities experience limited access to fruits and vegetables (FV) and may benefit from food environment interventions to increase the number of produce markets selling FV. Systems analysis is an innovative approach for informing policy, systems, and environmental (PSE) change interventions for the food retail environment. However, there has been little research. This study describes a new decision-making model that optimizes placement of new FV markets in a rural community in Texas based on combinations of three intervention factors: recommended driving distance to nearest produce market, service capacity of new and existing FV markets, and financial resources. Models estimated the potential effects of three intervention outcomes: the number of new FV markets, the ratio of fast food outlets to FV markets, and population coverage. Secondary sources of data were used in the models. The analysis tested 27 different interventions and compared effects to a benchmark. The smallest increase in population coverage or the local population’s access to FV was 19% compared to benchmark, while other interventions increased access to 100%. Models showed that the largest relative gain in access to FV, 29% to 37% for the local population, was at a lower level of financial resource availability ($1516171819-20,000). Findings provide evidence for the potential effects of food environment changes for one rural Texas community. Stakeholders can generate insights to inform context-specific decisions about their communities. In addition, this new decision-making model can be adapted for other communities to support PSE change interventions for nutrition.

## Introduction

Rural communities have unique challenges such as low population density, high poverty, and limited infrastructure that contribute to disparities in access to healthy foods [1,2]. Access to food is considered an important environmental determinant of individual dietary behavior [3]. While rural communities are not all the same [4,5], generally rural residents have a lower healthy food intake and a higher burden of diet-related diseases such as obesity, diabetes, and cardiovascular disease [6–8].

Improving access to healthy food options is a complex problem [2,3]. Five key dimensions are highly relevant in attempting to find solutions: a) accessibility (the location of food outlets and ease of transportation to food outlets) [9], b) availability (adequacy of supply of food in terms of presence or type of food outlet or qualities of the foods offered) [10], c) affordability (relative value or worth) [10], d) acceptability (attitudes and preferences of consumers for food outlet features) [11], and e) accommodation (features that meet consumer need, like a food outlet accepting food assistance benefits as payment) [11]. Healthy food-store interventions (HFIs) have demonstrated limited success due to challenges in design and implementation [12,13]. HFIs aim to encourage healthier purchases or discourage unhealthy ones. Most of the HFIs reported in the literature have been implemented in urban settings and have been designed to improve access at the *store level* by targeting in-store availability, affordability, acceptability, or accommodation with various strategies [12,13]. There have been far fewer interventions to improve access at the *community level* (e.g., by targeting accessibility to or availability of food outlets in the local food environment), especially for rural communities. As such, there is an opportunity to apply innovative methods to design interventions that can improve access to healthy foods in rural communities.

This study presents a new decision-making model that optimizes placement of new produce markets selling fruits and vegetables (FV) based on combinations of intervention factors (i.e., recommended driving distance to nearest produce market, service capacity of existing and new FV markets, and financial resources available to open new FV markets) and intervention outcomes (i.e., proportion of local population with access to FV, number of new FV markets, and ratio of fast food outlets to FV markets). Specifically, the model informs how many and where to place new FV markets that will guarantee access within a specified driving distance for most of the population in a community. The model aligns well with the RE-AIM (Reach Effectiveness Adoption Implementation Maintenance) planning and evaluation framework in that it can be used to inform community-based health program selection and design when there is limited data on effectiveness, sustainability, and implementation, and its flexible design allows for adaptation to various community contexts [14].

## Methods

**Fig 1:**
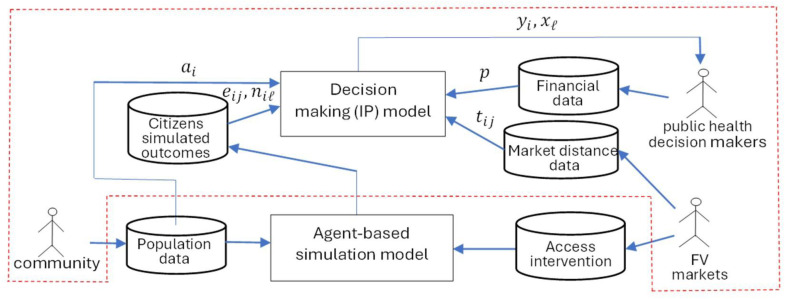
Framework for research study with data sources, models, and performance indicators for stakeholders.

The decision-making model proposed here takes the form of an integer program, constructed to work in conjunction with an existing agent-based model [15] that is designed to capture the effect on access to fruits and vegetables (FV) for local residents for one rural community in Northwestern Texas over a specified time period. Fig 1 illustrates the framework of the decision-making model. The red dotted line is used to highlight the data and actors. The cylinder symbols included in Fig 1 are the parameters or data inputs for the decision-making model. The outputs from the agent-based model were used as input parameters to the decision-making model. The purpose of the decision-making model was to optimize financial decisions in terms of the number and placement of new FV markets. Residents’ simulated outcomes, financial resources, and FV market distance data were included as inputs in the decision-making model. Population data included the number of residents per neighborhood. Market distance data per neighborhood included distances from each neighborhood to existing and potential new FV markets.

### Agent-based model

The new decision-making model, presented in this manuscript, is built on the agent-based model published by Katapodis, Zhang [15], which modeled the impact of accessibility on residents’ produce intake (i.e., dietary intake of fruit and vegetable) for one rural community in Northwestern Texas. The decision-making model considers multiple available locations (i.e., empty spaces in commercial buildings) in the community as potential locations to open new FV markets. In addition, the model accounts for the service capacity of the available locations (i.e., quantity of produce available for purchase) and budget constraints (i.e., financial resources needed to open new FV markets). Given those input factors, the model generates the recommended number and placement for new FV markets and the impacts of those decisions on the local population’s access to FV.

### Decision-making model for number and placement of New FV markets

The lead author (E.P.) created a new decision-making model using Microsoft® Excel and the add-in OpenSolver, which is an open-source tool for linear and non-linear optimizations (https://opensolver.org). The decision-making model was a resource allocation model, specifically an integer programing model. The model optimizes location for new FV markets and measures the impacts of improved access for a rural community. In other words, the model decides how many and where to place new FV markets that will guarantee access within a specified driving distance for most of the population.

To create the base model, sets, parameters, and decision variables were specified. The base optimization model included several constraints that varied depending on the intervention scenario to be modeled. The following equations were included in the base model. Equation 1 that maximized access to FV markets in a rural community. Equation 2 that modeled the constraint of a community being within a specified distance of an existing or new FV market. Equation 3 that limited the ratio of fast food outlets versus existing and new FV markets. Equations 4 and 5 were used to decide if a new FV market should be opened at a specified location. Equations 6 and 7 checked if a specific neighborhood was covered by either an existing or proposed new FV market. The base model was relevant for answering questions such as “What is the impact of considering different driving distances to define the residential food environment when selecting the optimal locations for new FV markets?” However, additional constraints were required to determine the impact of considering varied service capacity levels in the placement of new FV markets. Three equations were added; Equations 8–10 considered market factors like demand of FV and availability of FV within a community. Finally, Equation 11 was added to consider financial resource limitations of establishing a new FV market at an eligible location. Details about the decision-making model are discussed next.

Let I denote the set of neighborhoods in the city indexed i∈I and let set J denote the current markets in the community that sell fruit and vegetables, indexed j∈J. Set ℓ∈L represent empty commercial spaces in buildings available to rent or purchase, which can be a potential location for a FV market. Set K denotes the current fast food outlet in the community, indexed k∈K. The following three sets are coverage sets that identify locations for which the driving distance (d) is within a predetermined driving distance (S) from a destination location. Set $B_{i}$ represents the current FV markets (j) located within a predetermined driving distance S from neighborhood $i,B_{i}=\left\{(i,j)|dij\leq S\right\}$$N_{i}$ represents the current empty commercial buildings (l) available for rent and located within a predetermined driving distance (S) from neighborhood i, $N_{i}=\left\{\left(i,\text{l}\right)|di\ell \leq S\right\}$ $M_{i}$ represents the current fast food outlets (k in the community located within a predetermined driving distance S from neighborhood $i,M_{i}=\left\{(i,k)|dik\leq S\right\}$

The following parameters model important characteristics of the proposed model. Parameters nil, eij, and fik are binary in value and equal 1 when {(i,j)|dij≤S}, {(i,l)|diℓ≤S}, and {(i,k)|dik≤S}, respectively. If parameter niℓ=1 then neighborhood i has access to the new FV market located in ℓ. If parameter eij=1 then neighborhood i has access to an existing FV market located in j. If parameter fik=1 then neighborhood i has access to an existing fast food restaurant located in k. Parameter p models the monthly budget available to fund new FV markets and parameter cℓ represents the average operating cost per month of a FV market. Parameter $a_{i}$ represent the demand of FV per month for neighborhood i. Finally, parameters rj and rℓ represent the expected service capacity per month for existing and eligible new FV markets, respectively.

The decisions to be made by the model are represented by five binary decision variables: $x_{\ell }$, $y_{i}$, $u_{i\ell },v_{ij},\text{and}w_{ik}.$$x_{\ell }$ is binary and equals one if a new FV market is located ℓ. Decision variable $y_{i}$ equals one if community ihas a FV market located withing the predetermined driving distance S. Decision variable $u_{i\ell }$ equals one if community i is located withing the predetermined driving distance S of a new FV market ℓ. Decision variable $v_{ij}$ equals one if community i is located withing the predetermined driving distance S of an existing FV market ℓ. Finally, decision variable $w_{ik}$equals one if community i is located within the predetermined driving distance S of a fast food outlet k.

The optimization model can now be stated using the provided definitions for the sets, parameters, and decision variables. The number of constraints varies according to the research questions to be answered, however, five equations are always present in the model. The objective function (Equation 1), $\text{max}z=\sum i\in Ia_{i}y_{i}$, maximizes access to FV markets in a rural community. Equation 2, $y_{i}-\left(\sum {\mathrm{\backslash nolimits}}_{\ell }u_{i\ell }+\sum {\mathrm{\backslash nolimits}}_{j}v_{ij}\right)=\text{0},\forall i\in I$i is within S distance of an existing or new FV market. Equation 3, $\sum \mathrm{\backslash nolimits}k\in Kfik-\sum \mathrm{\backslash nolimits}j\in Jeij-\sum \mathrm{\backslash nolimits}\ell \in Lni\ell x\ell =R_{i},$ is the second constraint and limits the ratio of fast food outlets versus existing and new FV markets. The ratio is captured by parameter $R_{i}$. Equation 4, $Mx\ell \geq \sum {\mathrm{\backslash nolimits}}_{i}u_{i\ell },\forall \ell \in L$ equation 5, $x\ell \leq \sum {\mathrm{\backslash nolimits}}_{i}u_{i\ell },\forall \text{l}\in L$ℓ. Equation 6, $v_{ij}\leq e_{ij},\forall i\in I,\forall j\in J,$ equation 7, $u_{i\ell }\leq n_{i\ell },\forall i\in I\ell ,\forall \text{l}\in L,$i is covered by either an existing or proposed new FV market.

Equations 1–7 represent the “Base Model.” The “Base Model” is useful to answer many public health questions such as, *what is the impact of considering different driving distances to define the residential food environment when selecting the optimal locations for new FV markets?*. However, additional constraints must be added to the model to study *the impact of considering different service capacity for FV markets when selecting the optimal locations for new FV markets.* To study service capacity, three additional constraints were added to the “Base Model”. Equation 8, $\sum_{i}av_{ij}\leq rj,\forall j\in J$equation 9, $\sum {\mathrm{\backslash nolimits}}_{i}au_{i\ell }\leq r\ell ,\forall \text{l}\in L$ Equation 10, $\sum {\mathrm{\backslash nolimits}}_{j}v_{ij}+\sum {\mathrm{\backslash nolimits}}_{\ell }u_{i\ell }\leq \text{1},\forall i\in I$i.

To examine the impact of financial resources limitations, the model includes equation 11, $\sum {\mathrm{\backslash nolimits}}_{\ell \in L}c\ell x\ell \leq p$. A binary decision variable xℓ determines if a new FV market is sited at node ℓ and cℓ establishes the cost of establishing a new FV market in eligible market location ℓ. The right-hand side of the inequality (≤) establishes the maximum budget p availability to open new FV markets in the community.

The decision-making model is designed to be adaptable to various communities and can incorporate updated demographic and economic inputs. Parameters such as financial resources and the cost of establishing new FV markets can be adjusted to reflect current market conditions and funding availability. Similarly, the expected service capacity for existing and eligible new FV markets can be modified based on recent per capita dietary intake data and local population sizes. The model also utilizes population data and market distance data as inputs, allowing for updates to reflect current demographic and geographic information of a specific community.

### Intervention factors

The model considered how three different intervention factors, or constraints, affected intervention outcomes related to FV accessibility. The three intervention factors were: 1) recommended driving distance to nearest FV market; 2) financial resources required to open new FV markets; and 3) service capacity of the existing and new FV markets. Each intervention factor had three levels (i.e., low, normal, high). S1 Table provides more information on defining levels of intervention factors. The levels for recommended driving distance to nearest FV market was based distances of 1, 2.5, and 5 miles [16,17], where 5 miles represent the benchmark for rural residents. For financial resources, the benchmark was $20,000 per month, which was based on the USDA Farmers Market Promotion Program that provides up to $250,000 per year [18]. The model considered two additional levels ± $5,000 per month. The levels of low, normal, and high corresponded to monthly funding of $15,000, $20,000, and $25,000. The benchmark for service capacity of new FV markets was based on per capita dietary intake of FV [19] and local population size [20]. The model considered three market service capacity at low, normal, and high levels that provided 20,000, 40,000, and 80,000 servings of fruits and vegetables per month, respectively. Because there were interventions based on three different driving distances, each with three levels of financial resources, and three levels of service capacity, the study modeled a total of 27 intervention scenarios.

### Intervention outcomes

The model estimated effects for three different intervention outcomes: 1) population coverage; 2) number of new FV markets; and 3) ratio of fast food outlets to FV markets. The *population coverage* provides an estimate of the percentage (%) of the population that would have access to FV if the recommended number of new FV markets were established in the community. The *number of new FV markets* provides an estimate of the number of new FV markets to satisfy the community needs. The *ratio of fast food outlets to FV markets* provides an estimate of the number of fast food outlets per FV market in each neighborhood after implementation of the new FV markets. This study used multiple outcomes based on the heterogeneity in the literature [3,21,22], and prior research has shown that areas with limited access to supermarkets often had a higher concentration of fast food and convenience food outlets food [23,24]. S1 Table provides information about selecting ratio of fast food outlet to FV markets as an outcome.

The study does not involve the use of information about living humans through interaction or intervention or obtaining identifiable private information. As such, the study does not constitute human subjects research and does not require institutional review board approval at the institutions involved in the study.

## Results

The study area for the rural community is shown in S1 Fig. Using this decision-making model, Table 1 presents results of varying three different intervention factors to model three different intervention outcomes related to improving access to FV. The first intervention benchmark represented the do-nothing alternative or a no treatment control in an intervention. When no new FV markets were added to the local food environment, 46.4% of the local population had access to FV markets and the ratio of fast food outlets to FV markets was eight.

**Table 1:** Potential effects of different produce market interventions based on recommended driving distance, financial resources, and service capacity for a rural community in Northwestern Texas.

Interventions (n = 27)			Outcomes		
Recommended driving distance to nearest FV market	Financial resources required per month to operate new FV markets	Service capacity of new FV markets	Proportion of population with access to FV (%)	Number of new FV markets (*n*)	Ratio of fast food to FV markets
Benchmark: 5 miles	Benchmark: $0	Benchmark: normal	46.4%	0	8
1 mile	$15,000	low	55.1%	1	2.3
		normal	61.7%	1	2.3
		high	69.1%	1	2.3
	$20,000	low	55.1%	1	2.3
		normal	61.7%	1	2.3
		high	69.1%	1	2.3
	$25,000	low	55.1%	1	2.3
		normal	61.7%	1	2.3
		high	69.1%	1	2.3
2.5 miles	$15,000	low	73.6%	1	6.7
		normal	87.2%	1	6.7
		high	79.2%	2	5.7
	$20,000	low	79.2%	2	5.7
		normal	100.0%	2	5.7
		high	100.0%	2	5.7
	$25,000	low	79.7%	2	5.7
		normal	100.0%	3	4.7
		high	100.0%	3	4.7
5 miles	$15,000	low	73.6%	1	7
		normal	87.2%	1	7
		high	100.0%	2	6
	$20,000	low	79.2%	2	6
		normal	100.0%	2	6
		high	100.0%	2	6
	$25,000	low	79.7%	2	6
		normal	100.0%	3	5
		high	100.0%	3	5

As the recommended driving distance increased, and other factors held constant, the proportion of local population with access to FV markets increased. Using a recommended driving distance to nearest FV market based on a 1-mile radius, the maximum population coverage was 69.1% and this was achieved by opening one new FV market. Using a 2.5-mile or 5-mile radius, the maximum population coverage was 100%, and this was achieved by opening two or three new FV markets. In terms of service capacity, nine intervention scenarios were observed for each level of recommended driving distance. The maximum population coverage when the FV market monthly service capacity is set to low, and with driving distance of 2.5 or 5 miles, was 73.6%. This was achieved by opening one new FV market. The maximum proportion of population coverage when the when the FV market monthly service capacity was set to normal was 100%. This was achieved with two new FV markets. At a service capacity of high, the model showed that the maximum population coverage of 100% could be achieved with the addition of two FV markets.

In terms of financial resources, twenty-seven intervention scenarios were considered. The maximum population coverage when the financial resources availability was set to low was 100%. This was achieved by opening two new FV markets with high service capacity. The maximum population coverage when the financial resources availability was set to normal was 100%. This was achieved by opening two new FV markets at high service capacity. The maximum population coverage when the financial resources availability was set to is set to high was 100%. This was achieved by opening two or three new FV markets. Opening three markets reduced the ratio of fast food outlet to FV market ratio. Finally, assuming the lowest level of funding and service capacity, placement of one additional FV market within a 2.5-mile driving distance provided a maximum of 73.6% population coverage, which increased population coverage by 18.5%, compared to placement of the new FV market within a 1-mile distance (55.1%).

**Fig 2:**
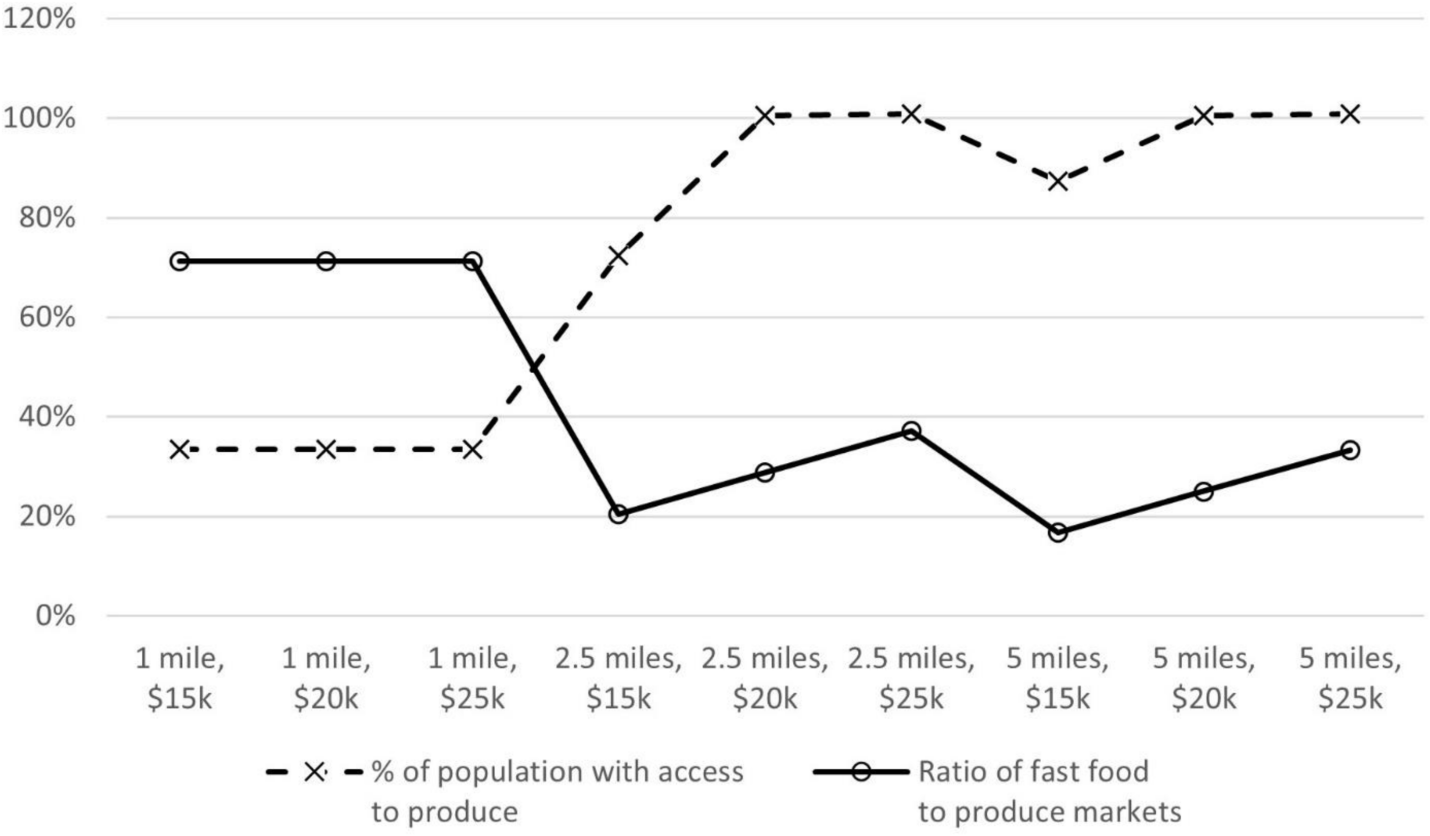
Improvements in intervention outcomes for different fruit and vegetable market interventions.

Fig 2 presents the percentage of improvement of all interventions when compared to benchmark. When the recommended driving distance to nearest FV market was set to 1 mile, and when financial resources availability equaled $15,000 per month, a 33% improvement in population coverage was observed relative to the benchmark. Notably, when driving distance was held constant at 1 mile, there was no percent improvement at higher levels of financial resources. When the recommended driving distance to nearest FV market was set to 2.5 miles, the amount of financial resources available impacted the percent of improvement for population coverage. For example, percent improvement increased from 72% to 100% when financial resources availability was increased from $15,000 to $20,000 per month. A similar pattern was observed when the recommended driving distance to nearest FV market was set to 5 miles, that is additional financial resources did not change the gains in population coverage.

In terms of the ratio of fast food (FF) to FV markets, Fig 2 shows that when the recommended driving distance to nearest FV market was set to 1 mile, a 71% improvement was observed when compared to the benchmark when financial resources availability to open new FV markets equaled $15,000 per month. However, the percentage of improvement did not change (i.e., 71%) for the ratio of FF to FV markets when comparing scenarios at a 1 mile driving distance. When the recommended driving distance to nearest FV market was set to 2.5 miles, the amount of financial resources impacted the ratio of FF to FV markets. For example, the percent improvement increased from 20% to 29% when financial resources availability was increased from $15,000 to $20,000 and from 29% to 37% when financial resources availability is increased from $20,000 to $25,000 per month. A similar pattern is observed when the recommended driving distance to nearest FV market was set to 5 miles.

### Policy implications

This study developed a new decision-making model for a local population in a rural community in Texas and explored how different food environment interventions might maximize a local population’s access to fruits and vegetables (FV). First, the simulated interventions produced quite large impacts. Regarding the estimated increase in access to FV, the smallest increase was 19%, that is population coverage increased from 46.4% with the benchmark to 55.1%. The 19% gain was based on a recommended driving distance of 1 mile, low financial resource availability of $15,000 per month, and a low service capacity of new FV markets and achieved by adding one new FV market. Other intervention approaches increased the proportion of the local population with access to FV to 100%. In real terms, a 19% increase in access to FV at the population level could be significant for public health. Second, the most impactful intervention approaches appeared to be the less intensive options, that is with relatively less financial resources or based on greater distances. For example, the most limited approach (i.e., with a $15,000 per month investment at the lowest level of financial resources, lowest service capacity, with recommended driving distance of 2.5 or 5 miles) produced a 27% increase (almost, from 46.4% to 73.5%) in population coverage. There was greater improvement in access to FV associated with increasing financial resources from $15,000 to $20,000 (20% to 29%), compared to higher levels or increasing from $20,000 to $25,000 (29% to 37%). With more financial resources or a greater service capacity, the model predicted that the entire community or 100% would have access, which represented a relative increase of nearly 55% in population coverage. The model that produced the greatest impact, relative to the control (or the do-nothing approach), was when the driving distance was 2.5 miles and with $15,000 of available funding. Third, interventions focused on improving access to FV may be more effective compared to interventions aiming to change the ratio of fast food (FF) to FV outlets. For example, this study demonstrated that the better-funded approaches at $25,000 per month with higher service capacities decreased in the ratio of FF outlets to FV markets (from a ratio of 8 to a ratio of about 5), but the largest decrease in the ratio used an optimal driving distance of one mile, which may not be practical for rural communities.

Related research related to improving food access has been efficiency-driven and used decision models to address well-defined technical problems [25–27]. For instance, Lien, Iravani [25] proposed an optimization model to solve the sequential resource allocation problem faced by a not-for-profit facility distributing donated food. Unfortunately, most of the published studies have drawn conclusions based on single attributes of the community food environment, such as proximity to the nearest supermarket or the density of FF outlets [26,27]. Since shopping and purchasing decisions are likely to be made based upon the market of food outlets that are available within a particular distance of travel, single attribute studies appear to be suboptimal [28–32]. Ratios of healthy to unhealthy food outlets and other multi-attribute approaches [33,34] to capture resident exposure are comparatively rare in the literature and warrant further investigation. This study makes an important contribution. Specifically, this study was unique in developing and testing a multi-attribute model to optimize the number and placement of new FV markets for a rural community in Texas. It is crucial to emphasize that the model is formulated as a general optimization model, designed for broad applications. The case study presented here, using data from a specific rural Texas community, serves primarily to illustrate the potential and capabilities of this model in informing food environment interventions. The model was created in Microsoft® Excel and an open-source add-in Open Solver, rather than a specialized software package. The model is available by request from the corresponding author. Given the accessibility of necessary local data, similar analyses can be performed in other communities. Decision makers, especially those with limited budgets, can use this model to tailor potential community food environment interventions to their communities and generate context-specific evidence for policy change.

Previously, review papers have summarized evidence for interventions that established new food retailers (i.e., farmers’ markets and mobile FV markets) and examined intervention effects on FV intake, but with mixed results [35–37]. For example, the Veggie Van in North Carolina was a mobile FV market that sold more affordable fruits and vegetables to residents living in socioeconomically disadvantaged communities of North Carolina [38]. Findings showed improvements in dietary intake of FV but limited success in changing access. However, this type of community-engaged intervention is resource-intensive. Further, there have been very few PSE change interventions with FV markets for rural communities. Additional research is needed to develop evidence-based recommendations for how to change community food environments in rural communities.

This study had several limitations. While decision-making models are advantageous for informing policy, systems, and environmental (PSE) change interventions for the community food environment [39], they cannot yet consider factors related to the consumer food environment. Prior research has shown the importance of in-store features, like preferred brands or products, prices, placement, and promotions on food purchasing [40]. Interventions aiming to positively change food environments, and support purchase of healthful foods like FV, must consider the factors, beyond accessibility, like affordability, acceptability, and accommodation [3,41,42], that are meaningful to potential consumers in rural communities, those living locally and those who travel to the food retail places from longer distances.

There are a few other limitations of this study. First, the decision-making model assumed that residents shopped within their neighborhood or local area, defined by a driving distance of up to 5 miles, but prior research has challenged that assumption [43,44]. Though this model considered some aspects of consumer choice by testing varied driving distances, this model is unable to consider food retail outlets beyond that area. Future research is warranted to integrate consumer behavioral factors into agent-based models. Second, this model was based on findings from an existing agent-based model, which was created using data from 2007 [28] and may not be as relevant for food purchasing in 2021. Third, this decision-based model was developed based on one rural community in Texas and, thus may have limited generalizability to other rural communities in Texas or other states in the U.S. However, given the complexity in developing agent-based models, this study still offers insights into the potential impacts of interventions to modify the community food environment.

## Conclusions

This study generated evidence for the potential effects of 27 interventions to the community food environment in one rural community in Texas and created a new decision-making model that may be customized to inform policy-making decisions in other communities. Overall, the most impactful interventions were less intensive with fewer financial resources or based on greater driving distances. Findings from this study have implications for policy and future research. Simulating multiple intervention conditions provides evidence that can be used to evaluate trade-offs in policy-making decisions and maximize community health impact. Given the scarcity of resources for testing multiple interventions, optimization models offer an efficient way to design interventions. Going forward, optimization models may be used to select the most promising food environment interventions for actual testing and implementation in specific communities. Future research directions may be to test combinations of interventions like new FV markets along with healthy food incentive programs [45], which help address affordability barriers and promote purchasing of FV. For example, the Gus Schumacher Nutrition Incentive Program (GusNIP) is a federally funded grant program that increases purchasing of fresh FV among income-eligible consumers (https://www.nifa.usda.gov/grants/programs/hunger-food-security-programs/gus-schumacher-nutrition-incentive-program). In Texas, the Double Up Food Bucks Texas program provides funding, through GusNIP, to match benefits for households participating in the Supplemental Nutrition Assistance Program (https://doubleuptexas.org/). Additionally, there are opportunities for future research to consider broad factors to support new businesses in a rural community, marketing campaigns promoting purchases of healthier foods from rural food retail venues [46], and recognition of greater needs, such as when a rural community might benefit from a supermarket and not an FV market, or other policy changes and environmental supports for food security [45].

## Supporting information

S1 Table Rationale for intervention factors and intervention outcomes in new decision-making model for number and placement of new FV markets. (DOCX)

S1 Fig Details for new decision-making model for number and placement of FV markets for a rural community in Northwestern Texas. (PDF)
