# Supplementary material for: Optimizing access to fruits and vegetables in rural communities: A decision-making model for the placement of produce markets
Source: PLoS One. 2025 Sep 26;20(9):e0331545. doi: 10.1371/journal.pone.0331545 (PMC12469377; doi:10.1371/journal.pone.0331545)
Supplement: S1 Table — (DOCX) [file pone.0331545.s001.docx]

**S1 Table. Rationale for intervention factors and intervention outcomes in new decision-making model for number and placement of new FV markets**

| **Intervention components** | **Rationale** | **Levels in model** |
| --- | --- | --- |
| Intervention factor |  |  |
| Recommended driving distance to nearest FV market | On average, rural residents are driving and 4.6 miles to a food outlet with a good variety of fresh and processed FV and 9.9 miles to the nearest supermarket (16). This study used a 5 mile radius as a benchmark. The low driving distance was based on the US Surgeon General recommended driving distance to FV markets or 1.6 km (1 mile), and the normal level was an intermediate distance between 1 mile and 5 miles. | - Low – 1 mile - Normal – 2.5 miles - High – 5 miles |
| Financial resources availability to open new FV markets | Although it is recommended that policymakers embrace a societal perspective and focus on total costs when making resource allocation decisions (48), in many cases the policy maker can consider only one component of cost. In this research, the benchmark value for financial resources availability factor is $20,000 per month, which is based on the USDA Farmers Market Promotion Program that provides up to $250,000 per year (18). The model considers two additional levels ±$5,000 per month. The purpose of those additional levels is to study the impact of funding limitations. Therefore, the levels of low, normal, and high correspond to monthly funding of $15,000, $20,000, and $25,000. | - Low – $15,000 per month - Normal – $20,000 per month - High – $25,000 per month |
| Service capacity of new FV markets | Each FV market needs sufficient physical space in the building for the fruits and vegetables, which are needed to meet the demand for fruits and vegetables of the local population. The authors considered three market service capacity levels (low, medium, and high) that will provide 20,000, 40,000, and 80,000 servings of fruits and vegetables per month, respectively. Levels were estimated using the expected number of fruits and vegetables consumed per person as reported by Caldwell et al. (19) and the size of the local population as reported by the U.S. Census (20). | - Low – 20,000 servings of FV per month - Normal – 40,000 servings of FV per month - High – 80,000 servings of FV per month |
| Fast food outlet to FV market ration | Some studies argue that living in potentially disadvantaged or unfavorable residential food environments in cities, such as a neighborhoods where fast food outlets concentrate, increases the risk of developing obesity through encouraging unhealthy food choices (21, 49). A study by Feng et al. (50) found evidence of higher body max index (BMI) among adults aged ≥ 45 years living within food environments defined by 1.6 km (1 mile) and 3.2 km (2 mile) buffers where at least one in four outlets was a fast food outlet. This result provides tentative support for actions to reduce concentrations in fast food outlets up to 2 km from home by providing healthier options. Furthermore, areas with limited access to supermarkets often have a higher concentration of fast food restaurants and convenience markets selling processed food (23, 24). The ratio of fast food outlets to FV markets addresses the impact of having a high concentration of fast food restaurants within the recommended driving distance to FV markets. |  |

This table presents additional information used to define the levels for each intervention factor.
