# Supplementary material for: Optimizing access to fruits and vegetables in rural communities: A decision-making model for the placement of produce markets
Source: PLoS One. 2025 Sep 26;20(9):e0331545. doi: 10.1371/journal.pone.0331545 (PMC12469377; doi:10.1371/journal.pone.0331545)
Supplement: S1 Fig — (PDF) [file pone.0331545.s002.pdf]

S1 Fig. Details for new decision-making model for number and placement of FV markets for a rural community in Nothwestern Texas.

Sets

- I denote the set of neighborhoods in the city indexed $i \in I$.
- Set J denote the current markets in the community that sell fruit and vegetables, indexed $j \in J$.
- Set $\ell \in L$ represent empty commercial spaces in buildings available to rent or purchase, which can be a potential location for a FV market.
- Set K denotes the current fast food outlet in the community, indexed $k \in K$.
- The following three sets are coverage sets that identify locations for which the driving distance (d) is within a predetermined driving distance (S) from a destination location.
    - Set B_i represents the current FV markets (j) located within a predetermined driving distance S from neighborhood $i, B_i = \{(i,j) | d_{ij} \leq S\}$.
    - Set N_i represents the current empty commercial buildings (ℓ) available for rent and located within a predetermined driving distance (S) from neighborhood i, $N_i = \{(i,\ell) | d_{i\ell} \leq S\}$.
    - Set M_i represents the current fast food outlets (k) in the community located within a predetermined driving distance S from neighborhood $i, M_i = \{(i,k) | d_{ik} \leq S\}$.

Parameters

The following parameters model important characteristics of the proposed model. Parameters $n_{i\ell}$, e_{ij}, and f_{ik} are binary in value and equal 1 when $\{(i,j) | d_{ij} \leq S\}$, $\{(i,\ell) | d_{i\ell} \leq S\}$, and $\{(i,k) | d_{ik} \leq S\}$, respectively.

- If parameter $n_{i\ell} = 1$, then neighborhood i has access to the new FV market located in ℓ.
- If parameter $e_{ij} = 1$, then neighborhood i has access to an existing FV market located in j.

- If parameter $f_{ik} = 1$, then neighborhood i has access to an existing fast food restaurant located in k. Parameter p models the monthly budget available to fund new FV markets and parameter c_ℓ represents the average operating cost per month of a FV market. Parameter a_i represent the demand of FV per month for neighborhood i.
- Parameters r_j and r_ℓ represent the expected service capacity per month for existing and eligible new FV markets, respectively.

Decision Variables

The decisions to be made by the model are represented by five binary decision variables: x_ℓ, y_i, $u_{i\ell}$, v_{ij}, and w_{ik}.

- Decision variable x_ℓ is binary and equals one if a new FV market is located ℓ.
- Decision variable y_i equals one if community i has a FV market located withing the predetermined driving distance S.
- Decision variable $u_{i\ell}$ equals one if community i is located withing the predetermined driving distance S of a new FV market ℓ.
- Decision variable v_{ij} equals one if community i is located withing the predetermined driving distance S of an existing FV market ℓ.
- Decision variable w_{ik} equals one if community i is located within the predetermined driving distance S of a fast food outlet k.

Optimization Model with Equations for Base Model and Modified Base Model

The optimization model was based on definitions for the sets, parameters, and decision variables. Equations 1-7 represent the Base Model.

- Equation 1 (the objective function), $\max z = \sum_{i \in I} a_i y_i$, maximizes access to FV markets in a rural community.
- Equation 2, $y_i - \left(\sum_\ell u_{i\ell} + \sum_j v_{ij}\right) = 0, \ \forall i \in I$, is the first model constraint and it verifies if community i is within S distance of an existing or new FV market.
- Equation 3, $\sum_{k \in K} f_{ik} - \sum_{j \in J} e_{ij} - \sum_{\ell \in L} n_{i\ell}\, x_\ell = R_i, \forall i \in I$, is the second constraint and limits the ratio of fast food outlets versus existing and new FV markets. The ratio is captured by parameter R_i.
- Equation 4, $Mx_\ell \geq \sum_i u_{i\ell}, \ \forall \ell \in L$, and Equation 5, $x_\ell \leq \sum_i u_{i\ell}, \ \forall \ell \in L$, are used to decide if a new FV market should be open at location ℓ.
- Equation 6, $v_{ij} \leq e_{ij}, \ \forall i \in I, \forall j \in J$, and Equation 7, $u_{i\ell} \leq n_{i\ell}, \ \forall i \in I, \forall \ell \in L$, check if neighborhood i is covered by either an existing or proposed new FV market.

To study service capacity, three additional constraints were added to the Base Model.

- Equation 8, $\sum_i a\, v_{ij} \leq r_j, \forall j \in J$, and Equation 9, $\sum_i a\, u_{i\ell} \leq r_\ell, \forall \ell \in L$, check that the expected demand for FV does not exceed the availability of FV.
- Equation 10, $\sum_j v_{ij} + \sum_\ell u_{i\ell} \leq 1, \forall i \in I$, determines which markets can serve every community i.

To examine the impact of financial resources limitations, additional equations were added.

- Equation 11, $\sum_{\ell \in L} c_\ell x_\ell \leq p$. A binary decision variable $x_\ell$determines if a new FV market is sited at node ℓ and c_ℓ establishes the cost of establishing a new FV market in eligible market location ℓ. The right-hand side of the inequality (≤) establishes the maximum budget p availability to open new FV markets in the community.

Details on sets, variables, and equations used in base models are variations on base model used to create new decision-making model.
